# Supporting Aging through Green Exercise (SAGE): Comparing the cognitive effects of outdoor versus indoor aerobic exercise in older adults with mild cognitive impairment—A 12‐week proof‐of‐concept randomized controlled trial

**DOI:** 10.1002/alz.091114

**Published:** 2025-01-09

**Authors:** Matt W Noseworthy, Nathan Wei, Sofia Grant, Jammy Zou, Eli Puterman, Todd C Handy, Teresa Liu‐Ambrose

**Affiliations:** ^1^ University of British Columbia, Vancouver, BC Canada; ^2^ Centre for Aging SMART, Vancouver Coastal Health Research Institute, Vancouver, BC Canada; ^3^ Djavad Mowafaghian Centre for Brain Health, Vancouver, BC Canada

## Abstract

**Background:**

Evidence from randomized controlled trials (RCTs) shows that aerobic exercise (AE) can benefit cognitive function among older adults with mild cognitive impairment (MCI). Growing research suggests that outdoor, natural (*i.e*., “green”) environments, compared with indoor or built environments, may augment the health benefits of exercise. However, the potential cognitive benefits of green exercise are unclear. We compared the effects of AE performed in an outdoor, green environment (O‐Ex) versus indoor AE (I‐Ex) on executive functions among older adults with MCI.

**Methods:**

A two‐arm parallel, single‐blinded, 12‐week proof‐of‐concept RCT. 72 community‐dwelling adults aged 65‐80 years with MCI were randomized (1:1) to either O‐Ex (n = 36) or I‐Ex (n = 36). Each arm involved two group‐based training sessions per week. Each session involved 10 min warm‐up, 40 min moderate‐intensity AE (progressing from 45%–75% of heart rate reserve), and 10 min cool‐down. We monitored exercise intensity using heart rate monitors and Borg’s 20‐point Rating of Perceived Exertion. AE consisted of outdoor walking in an urban forest (O‐Ex) or indoor treadmill walking or stationary cycling (I‐Ex). We measured working memory using the National Institutes of Health Toolbox (NIHTB) List Sorting Test and the Verbal Digits Forward and Backward Test. We measured set shifting and response inhibition using the NIHTB Dimensional Change Card Sort Test and Flanker Test, respectively. We performed a complete case analysis using a one‐way analysis of covariance to compare groupwise changes in executive functions (NIHTB corrected T‐score), with baseline outcome score and baseline Montreal Cognitive Assessment (MoCA) score as covariates. Overall alpha was ≤0.05.

**Results:**

Mean baseline MoCA score was 22.36 (SD = 2.55). Mean age was 74.08 years (SD = 3.92). There were 26 males and 46 females. The attrition rate was 12.5% (O‐Ex = 5; I‐Ex = 4). At 12 weeks, after adjusting for baseline outcome and baseline MoCA scores, O‐Ex had significantly better scores than I‐Ex for set shifting (estimated mean difference: 7.46; 95% CI: [2.69, 12.23]; p = 0.003) and response inhibition (estimated mean difference: 3.81; 95% CI: [0.61, 7.01]; p = 0.021), but not for working memory.

**Conclusions:**

Aerobic exercise performed outdoors in nature, compared with indoor aerobic exercise, may provide greater cognitive benefits among older adults with MCI.

